# *APOEε4* and risk of Alzheimer’s disease – time to move forward

**DOI:** 10.3389/fnins.2023.1195724

**Published:** 2023-05-19

**Authors:** Iliya Lefterov, Nicholas F. Fitz, Yi Lu, Radosveta Koldamova

**Affiliations:** Department of Environmental and Occupational Health, School of Public Health, University of Pittsburgh, Pittsburgh, PA, United States

**Keywords:** Alzheimer’s disease, risk, APOE, TREM2, transcriptional control, animal model

## Abstract

The inheritance of Apolipoprotein E4 (*APOEε4*) brings the highest genetic risk of Alzheimer’s disease (AD), arguably the highest genetic risk in human pathology. Since the discovery of the association, APOE protein isoforms have been at the center of tens of thousands of studies and reports. While, without a doubt, our knowledge about the normal physiological function of APOE isoforms in the brain has increased tremendously, the questions of how the inheritance of the *APOEε4* allele translates into a risk of AD, and the risk is materialized, remain unanswered. Moreover, the knowledge about the risk associated with *APOEε4* has not helped design a meaningful preventative or therapeutic strategy. Animal models with targeted replacement of Apoe have been generated and, thanks to the recent NIH/NIA/Alzheimer’s disease Association initiative, are now freely available to AD researchers. While helpful in many aspects, none of the available models recapitulates normal physiological transcriptional regulation of the human APOE gene cluster. Changes in epigenetic regulation of *APOE* alleles in animal models in response to external insults have rarely been if ever, addressed. However, these animal models provide a useful tool to handle questions and investigate protein–protein interactions with proteins expressed by other recently discovered genes and gene variants considered genetic risk factors of AD, like Triggering Receptor expressed on Myeloid cells 2 (*TREM2*). In this review, we discuss genetic and epigenetic regulatory mechanisms controlling and influencing *APOE* expression and focus on interactions of APOE and TREM2 in the context of microglia and astrocytes’ role in AD-like pathology in animal models.

## Introduction

Alzheimer’s Disease (AD) is the sixth leading cause of death in the United States. There are two forms of AD: early-onset or familial AD (EOAD), which develops before age 65, and late-onset AD (LOAD). EOAD is caused by autosomal dominant mutations in 3 genes – Amyloid Precursor Protein (APP), Presenilin 1 (PS1), and Presenilin 2 (PS2). The LOAD develops later in life, and in some individuals above 85, with no causative gene mutations known. LOAD cases account for more than 95% of all AD cases. An estimated 6.5 million Americans aged 65 and older were living with Alzheimer’s in 2022. Seventy-three percent are age 75 or older. The cost of Alzheimer’s and other dementias (ADOD) to the nation in 2022 was calculated at $321 billion, and by 2050, these costs could reach nearly $1 trillion. More than 11 million Americans provide unpaid care for people with Alzheimer’s or other dementias. In 2021, these caregivers provided more than 16 billion hours of care valued at nearly $272 billion. The financial burden on American society from ADOD is enormous.

In addition to the cognitive decline, there are two morphological hallmarks of AD: extracellular deposits of β-amyloid (Aβ) peptide, called amyloid plaques and intracellular neurofibrillary tangles of tau protein (Jack et al., 2018; Chen and Holtzman, 2022). While it has been established for more than 30 years now that the highest risk for LOAD is associated with a specific allele of *APOE* gene – *APOEε4*, other common gene variants have been added to a long and ever-increasing list of genetic risk factors of various significance (Pimenova et al., 2017; Sims et al., 2017; Ando et al., 2022; Holstege et al., 2022). Half of those are associated with immune response (Wes et al., 2016). Among those, rare variants of *TREM2*, expressed in microglia, are associated with a risk close to a risk associated with the inheritance of a single *APOEε4* allele (Wolfe et al., 2018b). Environmental exposures, lifestyle, diet, traumatic brain injury, and an array of comorbidities have been implicated in LOAD risk, early pathogenesis, and progression, as well (Rao et al., 2023). Unfortunately, none of the knowledge regarding the risk factors of LOAD has translated into early diagnosis of the disease or meaningful direction toward successful therapeutic strategies. In this review, we discuss genetic and epigenetic regulatory mechanisms controlling and influencing APOE expression and focus on interactions of APOE and TREM2 in the context of microglia and astrocytes’ role in AD-like pathology in animal models.

## *APOE* genotype and the risk of Alzheimer’s disease is the strongest genetic association in human pathology

APOE an extremely important and indispensable protein expressed in multiple tissues and organs. It is important to underline that no pathological condition or disease presents with the lack of APOE due to genomic deletion. Functionally, at a biochemical level, APOE provides a scaffold for and is an integral structural part of lipoproteins. In brain, APOE is secreted primarily by astrocytes and, unlike in the periphery, is the major apolipoprotein of High Density Lipoprotein (HDL)-like discoidal particles. These brain HDL-like particles do not contain APOA-I. Thus, the transport of cholesterol and phospholipids in the interstitial fluid and between neural cells highly depends on APOE. The transport of cholesterol and phospholipids is the major function of APOE. Therefore, in case of presumptive dysfunctional APOE, it is reasonable to expect, as a consequence, multiple disturbed molecular and cellular processes. The term dysfunctional APOE is poorly defined, however (Fazio et al., 1994). In AD nonmutated APOE, regardless of the isoform, retains its normal biochemical function.

In an excellent review published 23 years ago, R. Mahley and S. Rall concluded that “APOE plays a part in many processes beyond its traditional role in cholesterol and lipoprotein metabolism” (Mahley and Rall, 2000). A limitation of understanding *APOE* as a risk of AD is that it is impossible to disentangle the “traditional”/biochemical role of APOE in cholesterol metabolism and its role in AD risk and pathology. Human *APOE* and its *ε2/ε3/ε4* alleles have been associated with many diseases and pathological conditions, including nonpathological aging, and AD (Corder et al., 1993, 1994; Roses et al., 1994; Davies et al., 2014; Table 1). This supports the statement that the genetic risk conferred by *APOE* is related to a perturbed primary function of APOE isoforms: cholesterol and phospholipid transport and metabolism. This, inevitably, includes AD. Of course, it would have been naïve to explain all of the above phenotypes, particularly AD, only by the disturbed major biological effect of APOE – cholesterol and phospholipid transport.

**Table 1:** List of diseases where an association with *APOE* variants has been identified using different models and approaches.

Disease	Model/Approach	Conclusion
Carotid Atherosclerosis; progression of (Ma W. et al., 2022)	Logistic regression analysis	ε4 allele is independent risk factor for CAS in Han populations; the association is partly mediated through blood lipids.
Cerebral microbleeds (CMB) (Romero et al., 2014)	Framingham Original and Offspring cohort; MRI	Association of hypertension, CMB, low cholesterol and ε4
Combat-Related Posttraumatic Stress Disorder (Kim et al., 2013)	Clinician-Administered PTSD and Combat Exposure Scales with an assessment of the severity of alcohol use; logistic regression analysis.	APOEε2 allele operates as a susceptibility gene for combat-related PTSD, with the relationship between alcohol use and PTSD differing according to the APOEε2 carrier status
Coronary artery disease (Ashiq and Ashiq, 2021)	Meta analysis; ε2 vs. ε3 & ε4 vs. ε3.	ε4 allele appears as a significant genetic risk factor for coronary artery disease while the ε2 allele is beneficial to alleviate the CAD risk
Dementia with Lewy bodies (Bras et al., 2014)	GWAS	*APOE* genetic locus, driven by ε4 allele, is the strongest genetic risk factor for DLB
Exceptional longevity (Garatachea et al., 2014)	Each genotype and allele compared with all other genotypes and alleles	ε4 allele decreases the likelihood of reaching EL among individuals of different ethnic/geographic origins. ε2 favors EL, at least in the Italian and Japanese cohorts.
Frontotemporal lobar degeneration (Rubino et al., 2013)	Meta analysis; ε4 carriers vs. non-ε4 carriers; ε4 carriers vs. ε3 carriers	Evidence for an association between the ε4 allele and frontotemporal lobar degeneration.
Glomerular filtration rate (eGFR) (Coto et al., 2013)	Modified Diet in Renal Disease formula; multivariate logistic regression analysis	ε4 allele is a genetic risk factor for impaired renal function among healthy elderly Spanish individuals
Incident dementia risk in late life and early-life educational attainment (Ma H. et al., 2022)	Cox proportional hazards models	Higher educational attainment in early life may attenuate the risk for dementia, particularly among people with high genetic predisposition (one or two ε4 alleles).
Incident MCI and Physical Activity (Krell-Roesch et al., 2016)	Cox proportional hazards models; Prospective cohort study	Higher risk of incident MCI in ε4 carriers compared to ε4 non-carriers who reported physical activity
Late life depression (Yen et al., 2007)	Questionnaire; multinomial logistic regression analysis	ε4 allele may be correlated with severe depression in the elderly
Metabolic syndrome (Carty et al., 2014)	Meta analysis	*APOC1/APOE/TOMM40* significantly associated with MetS components overall
Mood disorders # Cardiometabolic disease (Amare et al., 2017)	Meta-GWASs;	*APOE4* in a group of 24 pleotropic genes participates in biological mechanisms of mood disorders and cardiometabolic diseases
Non pathological cognitive aging (Davies et al., 2014)	GWAS	Both *APOE* (rs429358 and *TOMM40* (rs11556505; as loci that were associated with cognitive agin)
Parkinson’s disease (Mata et al., 2014)	Cognitive test; Verbal learning	ε4 is a predictor of cognitive function in PD
Primary progressive aphasia and speech apraxia (Josephs et al., 2014)	Confirmed PPA, *APOE* genotyping and PiB PET	ε4 increases the risk of β-amyloid deposition in PPA and progressive speech apraxia but does not influence regional β-amyloid distribution or severity
Rheumatoid Arthritis (Chen et al., 2020)	CVD risk association	Lower risk for CVD in patients with ε2ε3 genotype compared to ε3ε4
Schizophrenia (Allen et al., 2008)	Meta analysis; ε4 vs. ε3	*APOE* in a group of 16 genes shows significant effect
TBI and deposition of Aβ (Nicoll et al., 1995)	Histological examination, *APOE* genotyping	The frequency of ε4 in those individuals with Aβ deposition following head injury (0.52) is higher than in most studies of Alzheimer’s disease
Vascular dementia (Chuang et al., 2010)	Cox proportional hazards models	*ε4* allele is associated with an increased risk of Vascular Dementia in a dose dependent fashion
Vascular disease (Koopal et al., 2015)	ε2 heterozygosity vs. all other Е genotypes	Distinct modifier effect of *APOEε4* on the relation between adiposity and lipids

The statements in the third column are concise conclusions related, as much as possible, mostly to APOE, in case the association includes other genes or conditions. The Table should be considered incomplete since (1) it does not include studies published in 2023, and (2) numerous studies that have confirmed already established associations between APOE and CVD.

Numerous proposed hypotheses explain how APOE isoform-specific differences might increase the risk of AD, ranging from neurotoxic effect based on domain interaction, binding to, deposition and clearance of Aβ, differential lipidation of isoforms, and neurotoxic and neuroprotection effects. More than 30 years since the association of *APOEε4* and the risk of AD has been established (Corder et al., 1993), we still do not know how exactly the role of APOE coded by *APOEε4* allele (APOE4) in cholesterol and lipoprotein metabolism – normal or disturbed, translates into an increased risk of AD. Nevertheless, in the last 2 years, several reviews and research papers have been published pointing to aspects of APOE biology that might be worth considering in terms of deciphering the role of APOE as a risk factor of AD and even designing new therapeutic strategies (Parhizkar et al., 2019; Chen and Holtzman, 2022; Li et al., 2022; Lindner et al., 2022; Martens et al., 2022; Raulin et al., 2022). In an *in-vitro* experimental system, Lindner et al. (2022) explored isoform-specific lipidation and revealed different lipidation pathways. While ATP Binding Cassette transporter A1 (ABCA1)-regulated APOE lipidation is isoform independent and cholesterol-rich HDL-like lipid particles are secreted by astrocytes, in stress-associated conditions, assembling and secretion of triacylglycerol-rich lipoproteins is boosted by the APOE4 isoform. The authors showed that APOE4 was a strong triacylglycerol binder and thus had a reduced capacity to clear toxic fatty acids from the extracellular milieu. Since 2003 (Koldamova et al., 2003), the importance of Nuclear Receptor’s Liver X Receptors/Retinoid X receptors/ABCA1-APOA-I/APOE (LXR/RXR-ABCA1-APOA-I/APOE) regulatory axis for normal/physiological function of APOE, and its relevance to AD pathogenesis in particular, has been demonstrated in tens of studies [for comprehensive reviews see Fitz et al. (2019), Koldamova and Lefterov (2007), Pahnke et al. (2021), Wolfe et al. (2018a), and Kim et al. (2009)]. The regulatory axis does not simply imply transcriptional regulation. While *ABCA1* is a primary LXR/RXR target gene, APOE and APOA-I are part of the axis because transcriptional upregulation of *ABCA1* and ABCA1-mediated lipidation of APOA-I and APOE are prerequisites for their stability, avoiding fast apolipoprotein degradation.

Perhaps the most comprehensive review of disturbed molecular, cellular, and pathophysiological processes in AD that might be associated with APOE4 has been published by researchers previously or currently affiliated with Mayo Clinic and Washington University at St Louis (Martens et al., 2022). Alike to Amyloid cascade hypothesis proposed in the late 90s [see J. Hardy for a comprehensive review and critical reappraisal (Hardy, 2009)], the authors coined the term “APOE cascade hypothesis” in the pathogenesis of AD and related dementias. Besides the excellent description of disturbed molecular and cellular processes during AD progression, Martens et al. propose a targeted engagement type of therapeutic strategy based on APOE biology and the physical properties of multimolecular complexes where APOE is an integral part. To be successful, these strategies should nevertheless consider that APOE is not a cause of AD as well as the role of APOE isoforms in development and normal CNS physiology.

## Expression of the Apolipoprotein E/C-I/C-IV/C-II gene cluster is highly regulated and differs in humans and mice

**Figure 1:**
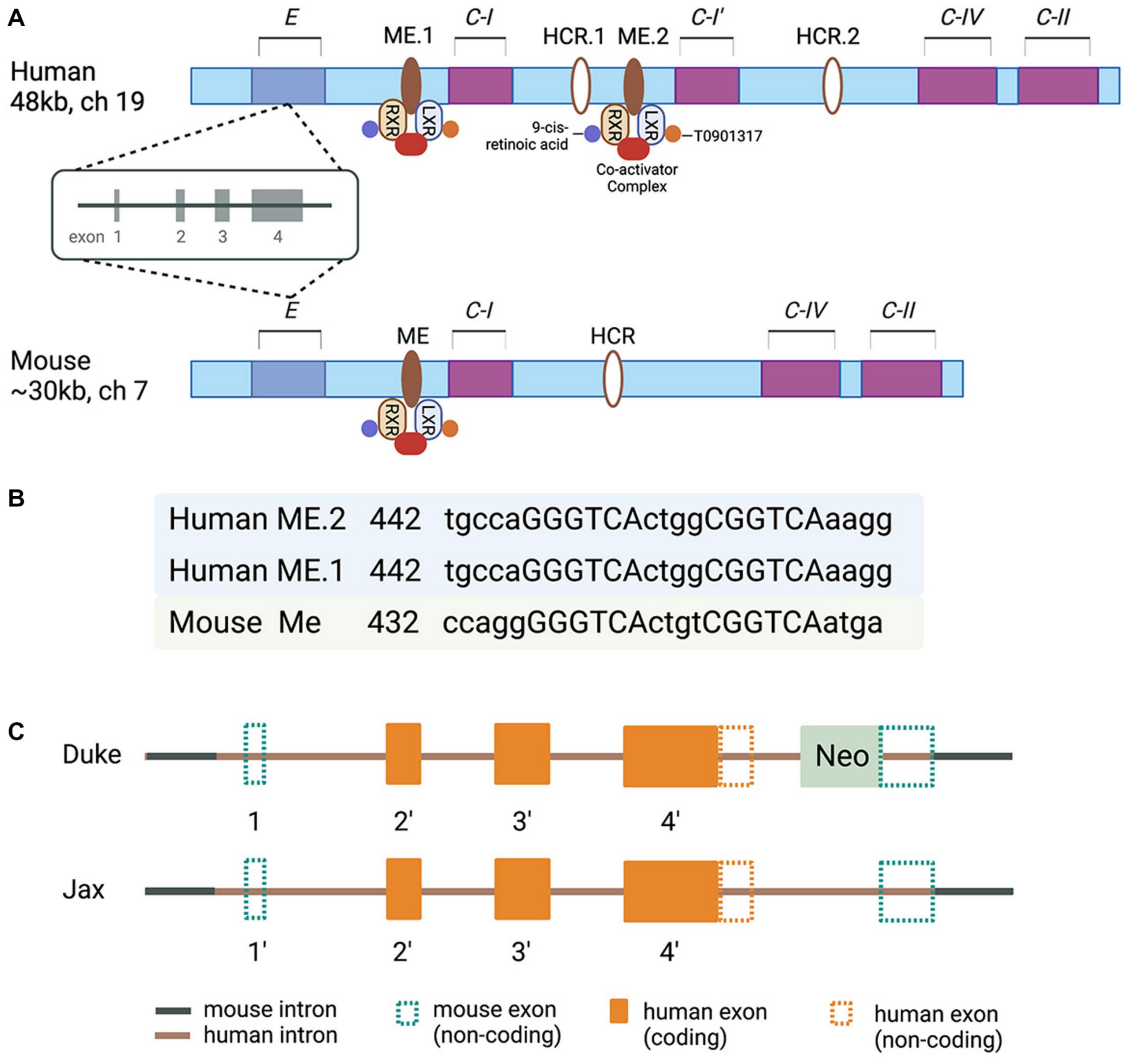
**(A)** Schematic of the human and murine APOE/C-I/C-IV/C-II gene clusters. Human cluster contains the *APOC-I* pseudogene. In the multienhancer regions downstream from human and mouse *APOE/Apoe* genes are located multienhancer elements ME1 in human and ME in mouse clusters. Human and mouse Hepatic Control Regions HCR.1 and HCR are located downstream of C-I. Human *APOC-I* pseudogene is indicated as C-I′; the pseudogene is a result of *APOC-I* duplication and does not exist in the mouse cluster. ME2 is a duplicated ME1 and exists only in humans. **(B)** The nucleotide sequences corresponding to putative LXR response elements with the 4-bp spacer within the human and murine MEs are shown as capital letters. The direct repeats are separated by 4-bp spacers. The locations of these sequences relative to the 5′  end of each ME are given. **(C)** A diagram of chimeric genes in TR APOE model mice generated at Duke (Sullivan et al., 1997) and Jax Labs (Foley et al., 2022). Detailed explanations are provided in the text.

Molecular clues to understand the regulated expression of Apolipoprotein E/Apolipoprotein C-I/Apolipoprotein C-IV/Apolipoprotein C-II (*E/C-I/C-IV/C-II*) gene cluster came from a series of reports based on studies conducted in J. Taylor’s and D. Mangelsdorf’s laboratories in the late 90s and the beginning of this century (Simonet et al., 1993; Allan et al., 1995, 1997; Shih et al., 2000; Grehan et al., 2001; Laffitte et al., 2001, 2003; Mak et al., 2002). The characterization of transgenic mice overexpressing individual genes of the cluster revealed diverse functions. However, the expression of all members of this apolipoprotein gene cluster is reported to be coordinately regulated by distal enhancer regions (Simonet et al., 1993; Allan et al., 1995). It was demonstrated that the regulatory sequences in the MultiEnhancers ME1 and ME2 downstream from the Apolipoprotein C-I (*APOC-I*) gene are transcription factors Liver X Receptor/Retinoid X receptor (LXR/RXR) Nuclear Receptor dimers canonical response elements (Mak et al., 2002; Figure 1). The regulatory sequences in the promoter regions of the human *APOE* gene cluster represent binding sites/response elements of additional transcription factors – APP-2, SP1, Estrogen Receptor, as well as transcriptional elements A, B, B1, and B2 (Smith et al., 1988; Jo et al., 1995; Paik et al., 1995; Bullido and Valdivieso, 2000). Furthermore, polymorphisms in the proximal promoter and the first intron of the *APOE* gene cluster (−1,019 to +407) affecting *APOE* expression were identified in the late 90s and early 2000s (Mui et al., 1996; Lambert et al., 1997, 1998a,b, 2000; Bullido and Valdivieso, 2000; Lumsden et al., 2020). Importantly, these polymorphic sites have been associated with a differential risk of AD (Artiga et al., 1998; Sims et al., 2017). However, the association of those polymorphic sites, sequence variability in the proximal promoter and ME1 and ME2, and the level of APOE protein in AD are not clearly understood. The data on the expression level of *APOE* RNA and the correlation with APOE protein level differ across the studies, and the question why remains unanswered. Importantly, in human postmortem brain, a CGI island that overlaps with exon four and downstream is highly methylated, and the methylation is altered in AD frontal lobe (Lee et al., 2020). However, the methylation level of *APOE* CGI correlates to the expression level of four known APOE transcripts, of which only one is full-length. Surprisingly, circRNAs, miRNAs, and truncated *APOE* transcripts constitute a significant part of the total *APOE* mRNA with higher expression in the AD frontal lobe. While the precise clinical significance of these variations in the amounts of RNA and methylation level of CGI in the *APOE* 3′-exon are not fully understood, the results of an increasing number of studies point to the regulatory role of epigenomic signatures and changes in epigenome associated with risk or clinical presentation of a variety of neurological disorders (Lee et al., 2020).

## The human APOE targeted replacement and other humanized mouse models

For the last 25 years or so, the availability of a mouse model with a targeted replacement of mouse *Apoe* gene (Sullivan et al., 1997) provided an opportunity to address a myriad of questions ranging from the role of APOE isoforms in AD pathogenesis to how successful variety of therapeutic strategies have been so far. The model has been used in multiple studies aiming to reveal the role of APOE isoforms in the response to traumatic brain injury, as well (Crawford et al., 2002, 2009; Castranio et al., 2017, 2018). Considering the differences between mouse and human APOE gene clusters, how complex the transcriptional control of human *APOE* is, the structure of the targeting construct (s), and the strategy to replace mouse *Apoe* in the TR APOE models (Sullivan et al., 1997, 2009) become important factors. An NIH/NIA initiative recently established the MODEL-AD (Model Organism Development and Evaluation for Late-onset AD) consortium to provide the scientific community with AD animal models of LOAD that better mimic human disease (www.model-ad.org). The consortium has created knock-in humanized coding and non-coding LOAD risk variants expressed at endogenous levels, including mice expressing all APOE isoforms (Oblak et al., 2020; Kotredes et al., 2021; Foley et al., 2022). The targeting constructs used to generate APOE TR at Jax (Foley et al., 2022) differ from those originally used at Duke (Sullivan et al., 1997). In the Duke model the 3′ homology arm of the targeting construct, manipulated according to the classic genetic engineering technology, is upstream of the mouse *Apoe* exon 4 and 3′ noncoding sequences. That means no human regulatory sequences downstream of the human *APOE* gene exist in the chimeric gene. The targeting construct used in the Jax APOE TR mouse model was generated using a technology called recombineering (Sharan et al., 2009), which allows engineering of large constructs (>100 kb) and recombination events without leaving any ‘footprints’ behind homologous recombination. There are two differences between the targeting constructs/chimeric genes used in the Duke and Jax models: (1) in the Duke model the distal part of human *APOE* 1st intron, followed by the entire downstream sequences and part of 3′ sequences downstream of exon 4, followed by a Neo cassette with a stop codon are inserted between *Sac I* recognition site downstream of mouse 1^st^ exon/intron junction, and *Pvu I* recognition site within mouse *Apoe* exon 4 (Sullivan et al., 1997, 2009). (2) in the Jax model, the chimeric gene retains part of human regulatory sequences upstream of the noncoding exon 1and the entire genomic sequence of *APOE*. There is no Neo cassette and downstream of human *APOE* 3′ noncoding sequences the chimeric gene retains a small part of mouse distal noncoding exon 4 (Foley et al., 2022; Figure 1). Neither of the chimeric genes has human regulatory sequences downstream of *APOE*. While this is hard to predict with certainty, most probably the expression levels and response to external stimuli/insults in TR mice will not differ, regardless of the model. The availability of Jax APOE TR mice to the AD research community and the opportunity to generate, examine and compare APOEε3/ε4 heterozygous mice to APOEε3/ε3 and APOEε4/ε4 mice is what makes the models hugely different. So far, the patent restrictions have not allowed the generation of heterozygous TR mice. We can speculate, however, that the expression of APOE isoforms, in the brains of Jax TR mouse models, does not recapitulate the transcriptional control and the regulation of the expression of APOE isoforms in the human brain. While this poses some questions about how good the models are at studying the risk of AD, we can assume that in a mouse model without overexpression of human APP, different protein–protein interactions, receptor-ligand interactions, and downstream intracellular and extracellular effects, replicate true physiological or pathophysiological conditions responsive to regulatory mechanisms at various stages of a neurodegenerative disorder, including AD. We believe these are the principal arguments and a justification to conduct *in vivo* experiments using animal models expressing different APOE isoforms where mouse *Trem2*, for example, is physiologically expressed, globally deleted by genetic engineering, or expressed as a mutant form shown to be associated with AD. We expect that animal models allowing the analysis of APOE in the context of gene–gene and gene–environment interactions will appear soon.

In APP transgenic mice expressing human APOE isoforms, APOE affects Aβ clearance and deposition in an isoform-dependent manner (Holtzman et al., 2000; Bales et al., 2009; Castellano et al., 2011; Kim et al., 2011), and APOE lipidation level is of significance (Fitz et al., 2012; Liao et al., 2018). Data from ABCA1 deficient mice (Hirsch-Reinshagen et al., 2004; Wahrle et al., 2004) and Tangier disease patients with non-functional ABCA1 demonstrated that in the absence of cholesterol efflux plasma APOA-I is virtually missing and APOE in plasma and brain is significantly reduced [reviewed in Oram and Vaughan (2000) and Koldamova et al. (2014)]. It has been shown independently by three groups that in APP transgenic mice, global deletion of *Abca1* decreases APOE lipidation and significantly increases amyloid deposition (Hirsch-Reinshagen et al., 2005; Koldamova et al., 2005; Wahrle et al., 2005). Recently, Fitz et al., using preclinical AD mouse models, demonstrated that APOE3 lipoproteins, compared to APOE4, prompted faster microglial migration toward injected Aβ, facilitated Aβ uptake, and abrogated damaging effects of Aβ oligomers on cognition (Fitz et al., 2021). Applying *in vivo* two-photon imaging, they showed that the APOE3 lipoproteins caused microglia to move faster toward Aβ and surround it, thus decreasing the spread of Aβ (Fitz et al., 2021). In tau mouse models of AD, expression of APOE4 has been shown to exacerbate tau-mediated neurodegeneration compared to mice with APOE3 expression, while the selective removal of astrocytic APOE4 is protective (Wang et al., 2021).

## TREM2: expression, function, and effects

### TREM2 – gene and protein structure

**Figure 2:**
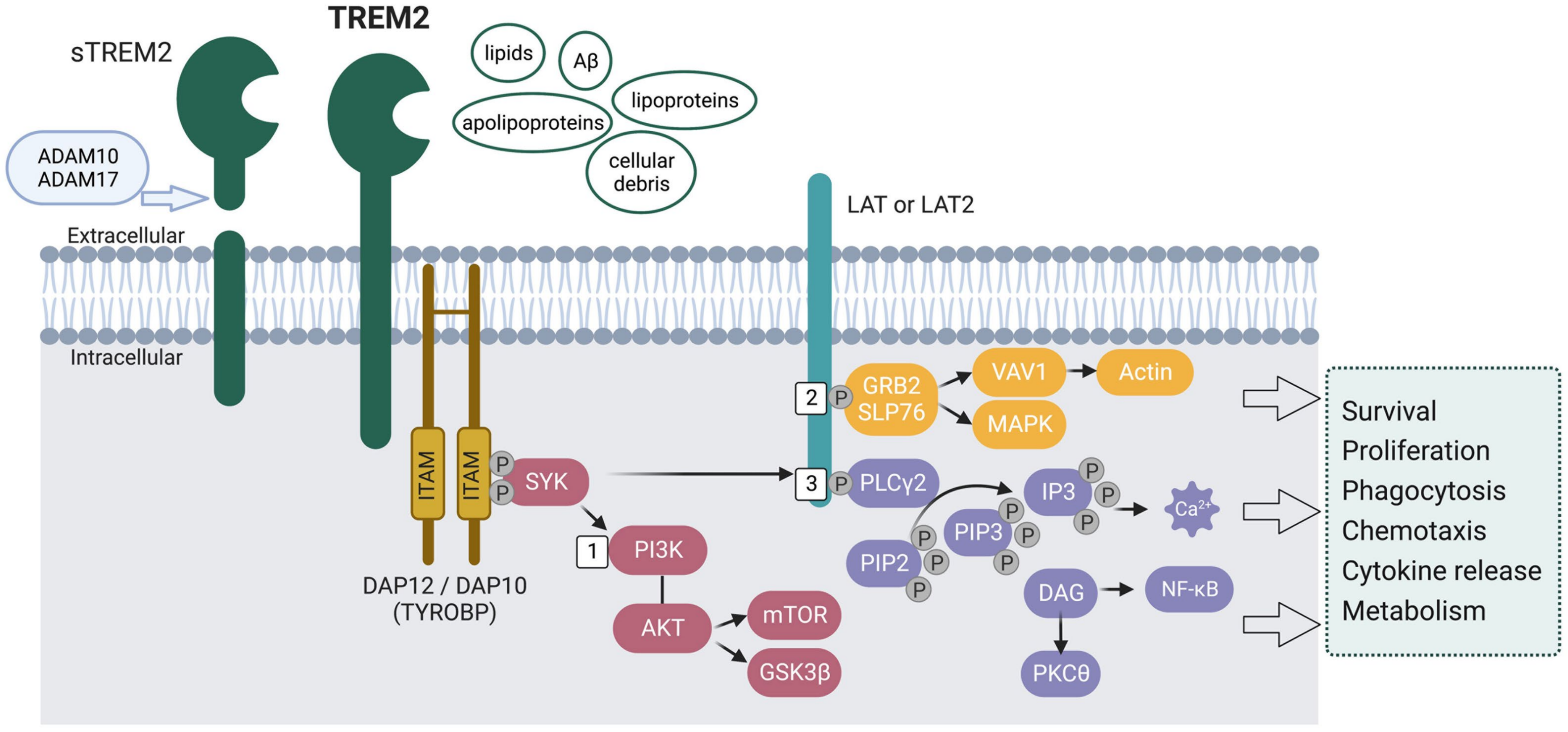
Schematic representation of the signaling pathways activated by the transmembrane receptor TREM2. The extracellular region of TREM2 is suggested to recognize and bind lipids, Aβ, lipoproteins, apolipoproteins, and cellular debris. Upon ligand binding, the cytoplasmic immunoreceptor tyrosine-based activation motifs (ITAMs) on DNAX-activating protein of 12 kDa (DAP12) recruit tyrosine protein kinase SKY, leading to activation of the phosphoinositide 3-kinase (PI3K) - AKT pathway and subsequent phosphorylation of linker for activation of T-cells family member 1 (LAT) and/or LAT2. Specifically, (1) The activation of PI3K leads to the activation of AKT, which in turn leads to the activation of mechanistic target of rapamycin (mTOR) signaling and the phosphorylation of glycogen synthase kinase 3β (GSK3β), resulting in GSK3β inactivation, stabilization of β-catenin, and cell cycling. (2) Upon phosphorylation of LAT/LAT2, adaptor molecules GRB2 and SLP76 are recruited, thereby activating the mitogen-activated protein kinase (MAPK) pathway and facilitating the recruitment of guanine exchange factors of the VAV family. This leads to the promotion of actin cytoskeleton rearrangement. (3) LAT/LAT2 also activates phospholipase Cγ2 (PLCγ2), which degrades phosphatidylinositol 4,5-bisphosphate (PIP2) into inositol 1,4,5-triphosphate (IP3) and diacylglycerol (DAG). This results in Ca^2+^ mobilization and NF-κB activation, respectively. These pathways have been found to affect microglia survival and functions, including phagocytosis, chemotaxis, cytokine release, and energy metabolism. Full-length TREM2 can be cleaved by a disintegrin and metalloproteinase domain-containing protein (ADAM10 and ADAM17), releasing soluble TREM2 (sTREM2).

TREM2, a member of the TREM family, is a cell surface transmembrane receptor with an extracellular Ig-like domain, a cytoplasmic tail, and a transmembrane domain [reviewed in Colonna (2023) and Wolfe et al. (2018b)] (see Figure 2). *TREM2* is expressed in cells of the myeloid lineage, such as microglia, osteoclasts, tissue macrophages, monocytes, and dendritic cells (Colonna, 2023). Most of the TREMs are evolutionarily conserved in mice and humans. Human *TREM2* is located on chromosome 6p21.1 in the *TREM* gene cluster near other *TREM* and *TREM-like* (*TREML*) genes: *TREML1*, *TREM2*, *TREML2*, *TREML3*, *TREML4*, and *TREM1* (Klesney-Tait et al., 2006). Mouse *Trem2* is located on mouse chromosome 17C in a cluster including *Trem1*, *Treml1, Trem2, Treml2*, *Trem3, Trem4, Treml4, Trem5, Treml6* (Colonna, 2023).

### TREM2 signaling

As shown in Figure 2, upon ligand binding, intracellular signals are conveyed through DNAX-activating protein DAP12 (12 kDA disulphide-bonded protein homodimer containing an immunoreceptor tyrosine-based activation motif), also known as TYROBP (tyrosine kinase-binding protein). The cytosolic immunoreceptor tyrosine-based activation motifs (ITAMs) on DAP12 will recruit the tyrosine protein kinase SKY, which leads to the activation of phosphoinositide 3-kinase (PI3K) - AKT pathway (Figure 2 #1) and phosphorylation of linker for activation of T-cells family member 1 (LAT) and/or LAT2, which recruit other signaling adaptors such as phospholipase Cγ2 (PLCγ2) (Figure 2 #3), and guanine exchange factor proto-oncogene VAV1 [reviewed in Colonna (2023)]. PLCγ2 further degrades phosphatidylinositol-3,4,5-trisphosphate (PIP3) to inositol trisphosphate (IP3) and diacylglycerol (DAG), thus mobilizing Ca^2+^. VAV1 induces actin remodeling that controls migration and adhesion (Figure 2 #2). All these signaling pathways affect the survival and proliferation of microglia (mTOR signaling), phagocytosis, and release of cytokines and chemokines (Wang et al., 2015; Wu et al., 2015; Raha et al., 2017; Ulland et al., 2017; Zhong et al., 2017; Zheng et al., 2018). Full-length TREM2 can be cleaved by disintegrin and metalloproteinase domain-containing proteins (ADAM10 and ADAM17, see Figure 2), thus releasing soluble TREM2 (sTREM2) (Wunderlich et al., 2013; Thornton et al., 2017). The exact biological and pathological role of sTREM2 is not clear, with some proposing it acts as a decoy receptor against TREM2 (Zhong et al., 2017) and others suggesting that sTREM2 plays an important role in promoting microglia survival and regulating inflammatory responses (Wu et al., 2015; Zhong et al., 2017). Overall, TREM2 expression and TREM2-induced signaling are essential in regulating microglia survival and proliferation.

### TREM2 ligands

The specific ligands that bind and activate TREM2 remain unclear. The extracellular region of TREM2 with an immunoglobulin domain binds many Gram-negative and Gram-positive bacteria and microbial products, such as LPS (Daws et al., 2003) and lipids [reviewed in Colonna (2023) and Kober et al. (2016)]. It has been shown that TREM2 binds phospholipids such as the phosphatidylserine on the surface of apoptotic cells (Poliani et al., 2015; Wang et al., 2015; Krasemann et al., 2017; Shirotani et al., 2019), lipoproteins including HDL and low-density lipoproteins (LDL) (Song et al., 2017). TREM2 also binds apolipoproteins, such as APOE (Atagi et al., 2015; Bailey et al., 2015; Yeh et al., 2016; Jendresen et al., 2017; Kober et al., 2020). TREM2 binds myelin lipids and participates in debris clearance and remyelination (Cantoni et al., 2015; Poliani et al., 2015; Nugent et al., 2020). It has also been reported that *in vitro* soluble TREM2 directly binds to Aβ oligomers (Lessard et al., 2018) and, at least *in vitro*, can facilitate Aβ degradation (Zhao et al., 2018).

### Regulation of Trem2 expression

Expression of *TREM2* is affected by inflammation; for example, pro-inflammatory molecules such as lipopolysaccharide (LPS) downregulate *TREM2* expression, and *in-vitro* anti-inflammatory molecules upregulated *TREM2* expression (Bhattacharjee et al., 2016; Liu et al., 2016; Zheng et al., 2016). *TREM2* expression is increased with the progression of neurodegeneration in AD patients (Perez et al., 2017) and mouse models of AD (Keren-Shaul et al., 2017; Krasemann et al., 2017) – traumatic brain injury (Castranio et al., 2017; Saber et al., 2017), Amyotrophic lateral sclerosis (Jerico et al., 2023), Parkinson’s disease (Liu et al., 2016), and post-stroke remodeling (Song et al., 2022). In terms of transcription factor regulation, a study by Daniel et al. (2014) demonstrated that the Retinoid X receptor (RXR) ligand LG268 increased RXR binding at a site upstream of the TREM2 locus, which regulates murine *Trem2* expression [also reviewed in Jay et al. (2017b)]. Treatment with another RXR agonist – bexarotene, enhanced the expression of TREM2 mRNA in the cortex of AD mice (Lefterov et al., 2015). A recent study demonstrated that HX600, a synthetic agonist for RXR-Nuss1 heterodimers, increased TREM2 immunoreactivity in an ischemic mouse model (Loppi et al., 2018).

### TREM2, amyloid deposition, and tau

The effects of *Trem2* deficiency on amyloid pathology have been studied in APP transgenic mice with different results based on the mouse model used and the stage of amyloid pathology. Wang et al. (Wang et al., 2015) were the first to report that *Trem2* deficiency significantly decreased the number of plaque-associated microglia in the 5XFAD AD mouse model, indicating that the proliferation of local microglia around the plaques is impaired, which other groups later confirmed (Yuan et al., 2016; Fitz et al., 2020). In their study, Wang et al. reported a significant increase of amyloid load in the hippocampus but not in the cortex at 8.5 months and no difference in amyloid load at an earlier age of 5XFAD mice (Wang et al., 2016).

Similarly, in APPPS1-21 model mice, there was no change in the amyloid pathology in the cortex but a significant decrease in the hippocampus of *Trem2*^−/−^ mice at 4 months (Jay et al., 2015). Interestingly, using the same AD mouse model at 8 months of age, Jay et al. showed an increase in 6e10 staining in the cortex and no changes in the hippocampus of *Trem2* deficient mice when compared to controls (Jay et al., 2017a) and concluded that in the early stages of amyloid deposition (2-month cortex, 4-month hippocampus) *Trem2* deficiency reduces both plaque number and size and at later stages of the disease *Trem2* deficiency increases plaque size and area. Using three different AD mouse models and employing high-resolution STORM imaging, Yuan et al. showed that *Trem2* deficiency increased the diffuse amyloid plaques associated with increased neuronal dystrophy (Yuan et al., 2016). What looks to be an undisputed feature of TREM2 is its role in microglia barrier around the amyloid plaques and in amyloid compaction (Wang et al., 2015; Yuan et al., 2016; Fitz et al., 2020; Meilandt et al., 2020; Wood et al., 2022). The conclusion from these studies is that the lack of microglia barrier in *Trem2* deficient mice impedes amyloid from forming dense plaques, thus allowing the spreading of more toxic Aβ oligomers.

The effect of Trem2 deficiency on tau pathology using tau mouse models is less clear and more complex. In P301S tau mice expressing mouse *Apoe* the loss of *Trem2* (Sayed et al., 2018; Leyns et al., 2019) or the expression of loss-of-function R47H variant (Gratuze et al., 2020) decreased brain atrophy and neurodegeneration as well as microgliosis compared to control mice expressing wild type *Trem2*. Surprisingly, the deletion of *Trem2* in the same P301S-tau model expressing human APOE4 isoform exacerbated tau-mediated brain atrophy (Gratuze et al., 2023). The most probable explanation for the observed discrepancies is that the mutated tau interacts differentially with human and mouse APOE. It is difficult to say the reason for these discrepancies without more experiments, including the expression of APOE2 and E3 isoforms and comparing their effect on tau pathology.

In 2017 using different models of neurodegeneration, several groups have identified and characterized the phenotype and transcriptomics of novel microglia type associated with neurodegenerative diseases called either Disease Associate microglia (DAM) (Keren-Shaul et al., 2017) or microglial neurodegenerative phenotype (MGnD) (Krasemann et al., 2017). DAM signature represents a unique set of genes that overlapped in different studies and includes the upregulation of genes such as *Apoe, Trem2, Clec7a, Axl, Lpl, Spp1, and Mpeg1* and downregulation of another set of genes termed “homeostatic” microglia (for example Tmem119, P2ry12) (Keren-Shaul et al., 2017; Krasemann et al., 2017; Sala Frigerio et al., 2019; Fitz et al., 2020). Keren-Shaul et al. determined that *Trem2* is required for the transition from one disease state to another, but the shift from homeostatic to DAM is Trem2 independent (Keren-Shaul et al., 2017). The finding that Trem2 deficiency suppresses the DAM program, including the expression of *Apoe*, was confirmed by other studies (Krasemann et al., 2017; Sala Frigerio et al., 2019; Fitz et al., 2020, 2021).

## APOE, TREM2, and AD

*APOE* and *TREM2* are part of a large group of GWAS-identified genes associated with AD risk with glial-specific expression (microglia and astrocytes) and are related to immune response (Villegas-Llerena et al., 2016). As noted above, at least *in vitro* TREM2 binds to APOE (Atagi et al., 2015; Bailey et al., 2015; Yeh et al., 2016; Lessard et al., 2018; Kober et al., 2020). In some studies, the R47H variant of TREM2 has markedly reduced binding between APOE and TREM2 (Atagi et al., 2015; Bailey et al., 2015; Yeh et al., 2016) and in others with no effect on their interaction (Lessard et al., 2018). In terms of the affinity of TREM2 to bind different APOE isoforms, two studies showed that APOE4 demonstrates a slightly higher affinity to bind TREM2 than the other two isoforms (Jendresen et al., 2017; Kober et al., 2020) but other – no significant difference between the isoforms (Atagi et al., 2015; Bailey et al., 2015; Yeh et al., 2017; Lessard et al., 2018). Regarding APOE lipidation, TREM2 was shown to bind lipidated and non-lipidated APOE. However, it is an open question if APOE lipidation affects TREM2 binding. Some studies found that their interaction is enhanced by lipidation (Yeh et al., 2016) and others to be slightly decreased by it (Kober et al., 2020). As noted above, bearing in mind the data from Tangier patients and *Abca1* knockout mice, it is possible that *in vivo* APOE does not exist in lipid-free form [reviewed in Oram and Vaughan (2000) and Koldamova et al. (2014)]. Thus, whether APOE lipidation has a role in TREM2 binding is more of a theoretical than practical significance.

Only a few reports examine the interaction between TREM2 deficiency and APOE isoforms *in vivo* either in APP transgenic models (Fitz et al., 2020) or in AD patients (Nguyen et al., 2020). Fitz et al. demonstrated that in APP mice expressing human APOE3 or APOE4 (APP/E3 and APP/E4), the lack of *Trem2* impaired microglia barrier in both isoforms but did not change steady-state plaque load (Fitz et al., 2020). However, *APOE* mRNA expression measured in plaque-associated microglia was significantly reduced by *Trem2* deficiency only in APP/E4 and not in APP/E3 mice, suggesting that APOE4 microglia respond differently to the absence of *Trem2* (Fitz et al., 2020). Since another DAM gene *– Clec7a*, was also significantly decreased in plaque microenvironment only in APP/E4 without *Trem2*, this might imply that DAM response to a challenge (in this case, amyloid deposition and *Trem2* absence) is less effective on an APOE4 background, thus preventing microglia around plaques to form a complete barrier (Fitz et al., 2020, 2021). A recent study by Nguyen et al. used single-nucleus RNA sequencing of postmortem human brain expressing *APOE* and *TREM2* variants and identified four distinct microglia clusters. One was the homeostatic microglia; the rest were “active” clusters designated based on different pathology and differentially expressed genes. Nguyen et al. established that one of the active clusters includes a subpopulation of CD163-positive microglia, which they named “amyloid-responsive microglia.” They determined that this cluster is enriched in APOE3/3 AD patients and is relatively depleted in cases with *APOE4* carriers and *TREM2* risk variants. Nguyen et al. proposed an amyloid-responsive microglia subpopulation primed to elicit an activated immune response and note the reduced response in APOE4 carriers. In a recent study, Fitz et al. showed that in mice injected with Aβ plus native APOE lipoproteins, there was a higher number of differentially expressed genes between WT vs. Trem2^ko^ if the mice were injected with APOE4 compared to APOE3, particularly genes associated with interferon signaling (*Ifit2*, *Ifi27l2a*, *Ifi207*, and *Axl*) and endocytosis (*Cd14*, *Cxcl16*, *Fth1*, and *Ifitm3*) (Fitz et al., 2021). Additionally, the lack of TREM2 decreases Aβ phagocytosis only by APOE4-treated microglia, thus suggesting that APOE4 lipoproteins compared to APOE3 are insufficient to resist TREM2 deficiency, particularly in the presence of Aβ (Fitz et al., 2021).

## Concluding remarks

It is hard to admit that 30 years after the discovery of *APOEε4* as the highest genetic risk of AD, the nature and the molecular and cellular mechanisms that materialize the risk are still poorly understood. Multiple and well-supported hypotheses have been proposed, pointing to various mechanisms explaining the risk conferred by APOE4. Most importantly, however, loss of function – decreased level of APOE4 and fast degradation of poorly lipidated Apolipoprotein E4, as well as gain of function – generation of neurotoxic fragments due to domain interaction and reduced stability of APOE4, seem to work in concert and gradually lead to dysbalanced Aβ clearance, facilitated tau fibrillation and higher order behavioral disturbances. It is possible that these two pathogenic pathways work in concert and should be addressed together. A better understanding of those seemingly distant pathways and the interaction of APOE4 with other signaling and regulatory molecules – ABCA1 and TREM2, for example, conferring an increased risk themselves, are very important and hopefully will point to reasonable and probably successful therapeutic strategies based on *APOEε4.* Until then, the ideas of replacing/eliminating APOE4, inhibiting its interactions at an ill-defined age, or ignoring the intervention time seem poorly substantiated. In this review, we cautiously, although briefly, emphasized the complexity of transcriptional regulation of the E/C-I/C-IV/C-II gene cluster and differences in regulatory, including epigenetic, mechanisms in humans and mice. These differences become even more important in APOE TR mice. An attempt to formulate a strong hypothesis on how the risk conferred by the inheritance of the *APOEε4* allele is materialized, based on the studies that use more or less complex or extremely complex animal models, is doomed to failure. The overwhelming controversies and inconsistencies in conclusions from otherwise perfectly conducted studies indicate the lack of the appropriate mouse model. Perhaps a model that includes *APOE*/*CI* part of *APOE* cluster with critical regulatory sequences is one promising option. The scientific community needs such a model/models to move further from square one – the inheritance of the *APOEε4* allele is the highest genetic risk of AD.

## Author contributions

IL and RK contributed to the conception of the review. YL wrote the first draft. IL, RK, and NF co-edited the manuscript. All authors contributed to the writing, have read and agreed on the final manuscript.

## Funding

This work was funded by the National Institute on Aging – National Institutes of Health, USA: R01AG06619, R01AG077636, R01AG075992, R01AG057565, and R01AG052978.

## Conflict of interest

The authors declare that the research was conducted in the absence of any commercial or financial relationships that could be construed as a potential conflict of interest.

## Publisher’s note

All claims expressed in this article are solely those of the authors and do not necessarily represent those of their affiliated organizations, or those of the publisher, the editors and the reviewers. Any product that may be evaluated in this article, or claim that may be made by its manufacturer, is not guaranteed or endorsed by the publisher.
